# Go Forth and Colonize: Dispersal from Clinically Important Microbial Biofilms

**DOI:** 10.1371/journal.ppat.1005397

**Published:** 2016-02-18

**Authors:** Priya Uppuluri, Jose L. Lopez-Ribot

**Affiliations:** 1 The Division of Infectious Diseases, Los Angeles Biomedical Research Institute at Harbor-University of California Los Angeles (UCLA) Medical Center, Los Angeles, California, United States of America; 2 Department of Biology and South Texas Center for Emerging Infectious Diseases, The University of Texas at San Antonio, San Antonio, Texas, United States of America; Geisel School of Medicine at Dartmouth, UNITED STATES

## Introduction

The past two decades have witnessed remarkable developments in the field of microbial biofilm research, with the implementation of advanced molecular technologies enabling in-depth understanding of the sessile lifestyle across a variety of systems and organisms. The keen focus on biofilms is justified mostly because of their significant clinical impact, which has prompted the need to explore, manipulate, and devise methods to both prevent biofilm development and treat established biofilms in diverse environments and clinical settings. Furthermore, in recent years, the study of how these surface-associated consortia of cells transition back to unattached, planktonic entities has become a theme of intense interest and scrutiny for microbiologists. Accumulating evidence indicates that escape or release of cells from a biofilm contributes to the success of the microbe as a pathogen. Here, we first provide a general overview of the differences in the biofilm life cycle between bacteria and fungi, with emphasis on medically important pathogens. Next, we elaborate on the recent advances in our understanding of biofilm dispersal and how the process can be harnessed for the clinical management of biofilm-associated infections.

## How Does the Biofilm Lifestyle Differ in Bacteria and Fungi?

The initial definition of biofilms as a community of microorganisms attached to a surface and embedded by an exopolymeric matrix has come to be recognized as an intricate developmental process that is complex and dynamic in nature. A shift from free-living growth to biofilm is triggered by environmental changes and involves multiple regulatory networks that control gene expression changes, leading to spatial and temporal reorganization of the microbial cell [1,2]. All biofilms, regardless of the species of the pathogenic microorganism that makes them, share several common features: production of exopolymeric substances (EPS), substantial resistance to killing by host defenses, and recalcitrance to antimicrobial drugs [2,3]. The sheer strength in numbers and the protective shield of EPS around the cells make most biofilm-associated infections difficult to treat and eradicate. Despite fundamental similarities, several distinct characteristics from both structural as well as developmental aspects exist between bacterial and fungal biofilms.

Bacterial biofilms typically develop in three distinct stages: attachment of cells to a surface, growth of the cells into colonies of sessile cells, and detachment of cells from the colony into the surrounding medium. In several gram-negative bacteria, attachment is reinforced by specific adhesins located on the bacterial cell surface or on cellular appendages such as pili and flagella [4]. Multiplication of bacteria on the surface is accompanied by concomitant synthesis of EPS that holds the bacterial cells together in a mass, firmly attaches the scaffold to the underlying surface, and contributes to antibiotic drug resistance, either by acting as a diffusion barrier or entrapping antimicrobial drugs, thereby preventing access to sessile cells within [3].

**Fig 1:**
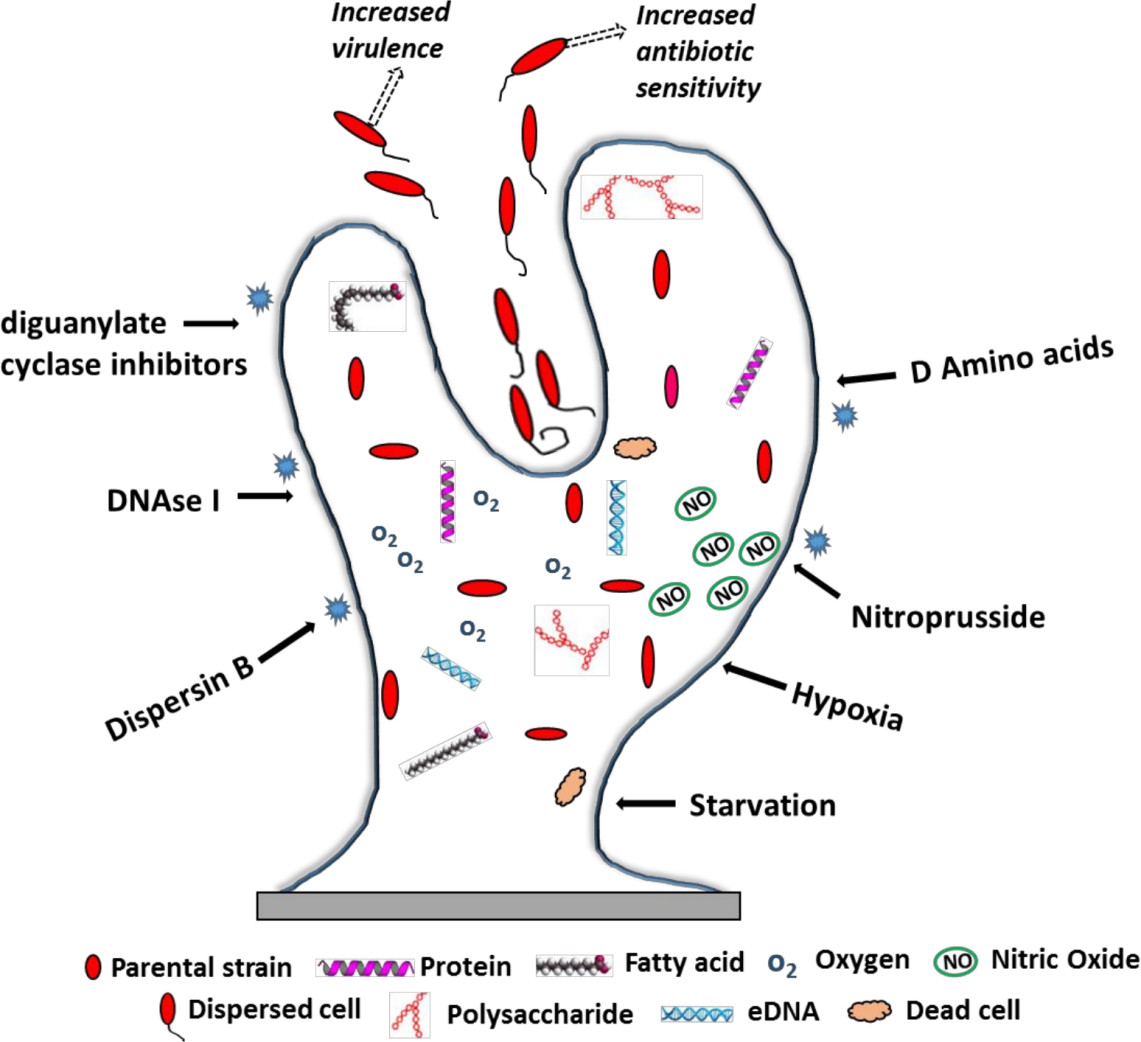
Biofilm dispersal in bacteria. Constituents present in a mature biofilm are described within the figure. Solid arrows refer to external triggers for biofilm dispersal. Dashed arrows indicate properties of the dispersed cells.

In several different bacteria, such as *Pseudomonas aeruginosa*, *Vibrio cholerae*, *Staphylococcus epidermidis*, *Streptococcus mutans*, *Streptococcus gordonii*, etc., mature biofilm colonies often adopt a peculiar “pillar and mushroom”-shaped formation that projects outward for hundreds of microns and is composed of millions of tightly packed cells [5]. As a consequence of their complex organizational structure, biofilms harbor numerous microenvironments differing in pH, oxygen, and nutrient availability, which lead to metabolic heterogeneity among cell populations in different locations of the biofilm (Fig 1). Finally, bacterial biofilms undergo detachment of cells and dispersal into the surrounding milieu—a process that proves integral for bacterial survival, propagation, and virulence. In bacteria, dispersal is frequently a terminal process, occurring right at the end of biofilm maturity and marking the end of the biofilm life cycle [4].

**Fig 2:**
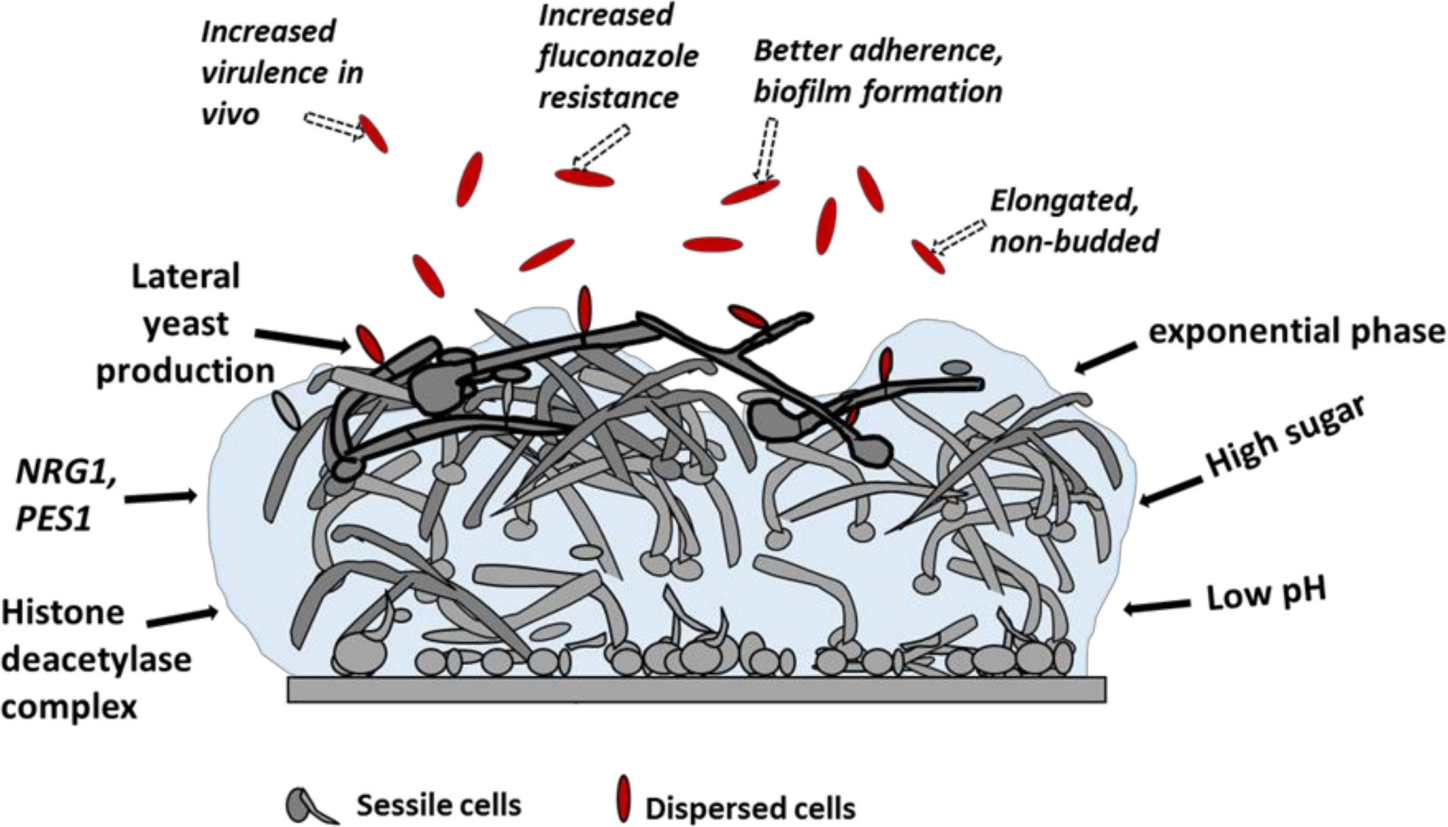
Biofilm dispersal in fungi (*C*. *albicans*). Biofilms are composed of basal parental yeast and germ tube cells, as well as a mesh of hyphal cells covered with EPS. Lateral yeast cells are composed of a large portion of elongated and virulent dispersed cells (dashed arrow), which are induced by a variety of external stimuli (block arrows).

Fungal biofilms largely follow a similar progression of events for biofilm development yet exhibit certain peculiarities. Like bacteria, biofilms are initiated upon adhesion of cells to a substrate where yeast cells or conidia adhere via specific cell surface adhesins, leading to subsequent transcriptional changes. Post-adherence, cells proliferate across the surface into filamentous forms known as hyphae. Presence of hyphae results in a much more cohesive and uniform biofilm as compared to the mushroom-like appearance typical of bacterial biofilms (Fig 2). While in many fungi, such as *Candida albicans* and *Aspergillus fumigatus*, hyphae are an integral part of biofilms [6], other fungi like non-*albicans Candida* spp. (i.e., *C*. *glabrata*) and *Cryptococcus neoformans* develop biofilms that are devoid of hyphae. EPS production is universal in fungal biofilms, and, similar to bacteria, the extracellular matrix plays a key role in biofilm integrity and contributes to antifungal drug resistance [7].

Dispersal in fungal biofilms has been a latent area of study and has recently been investigated only in *C*. *albicans*. Rather than an end stage process, release of cells occurs throughout the growth cycle and is composed of mostly unbudded yeast cells [8]. Apart from dispersal, *C*. *albicans* biofilms may also undergo a more dramatic massive detachment event, in which the entire biofilm can detach from the surface by hitherto unidentified mechanisms [9].

## What Are the Signals That Trigger Biofilm Dispersal?

Nutrition plays a key role in the biofilm dispersal process. In some bacterial biofilms, dispersal is activated in the presence of abundant nutrition. For example, in *Acenitobacter* sp. St GJ12, nutrient surplus induces rapid escape of cells from the biofilm, while carbon limitation causes the biofilms to get more compact [10]. *P*. *aeruginosa* biofilms also show a similar response, in which the carbon-dependent dispersion is transcriptionally regulated by the gene *BdlA2*, a chemotaxis regulator whose levels are affected by Cyclic-di-GMP (c-di-GMP), a common intracellular nucleotide [11]. In fact, a breakthrough in understanding of how cells sense their environment was made when it was discovered that levels of c-di-GMP governed the transition from biofilm to planktonic phenotypes in response to various environmental cues and cell–cell signals in bacteria [12]. Simply explained, increases in c-di-GMP levels favor biofilm growth, while decreases induce dispersal. However, regulation of c-d-GMP is extremely complex, involving the (redundant, yet less-understood) function of diguanylyl cyclases (DGCs) that synthesize c-di-GMP and the phosphodiesterases (PDEs) that degrade it. Besides nutrient sensing, exposure to hypoxia or to low levels of nitric oxide (NO) potentiates bacterial biofilm dispersal, and intriguingly, both these environmental triggers modulate their effects indirectly via c-di-GMP, making this nucleotide a central element for biofilm dispersal. NO is one of the most potent signals for biofilm dispersal in numerous medically important bacteria, including both gram-negative (*Escherichia coli*, *V*. *cholerae*, *Serratia marcescens*) and gram-positive (*S*. *aureus*) organisms (Fig 1) [13].

Studies investigating the environmental signals that modulate dispersion from fungal biofilms are still in nascent stages. To some extent, this has been examined in *C*. *albicans*, in which it was found that dispersal can be triggered by a carbon source, such as glucose, while other sources, such as maltose, galactose, and PBS (to induce starvation conditions), curtail dispersal to greater than 50-fold. The study was performed on biofilms developed under continuous flow of fresh medium in vitro, where it was observed that the richer the medium, the greater the dispersal [8]. Additionally, it was reported that pH of the growing medium also exerts an important effect on *C*. *albicans* biofilm dispersion, which is enhanced at acidic pH and decreased under alkaline conditions (Fig 2).

*C*. *albicans* biofilms are almost totally made up of hyphae, and hyphae are known to produce yeast cells from their subapical regions known as “lateral” yeasts. Most of the environmental cues for dispersion, as discussed above, are known to favor the yeast form over the hyphal morphology. The fact that dispersed cells are predominantly yeasts, despite the presence of a large population of hyphae in the mature biofilm, indicates that these cells emerge from the top-most hyphal layers of the biofilm. This idea was validated in the studies that showed *C*. *albicans* Pes1, a controller of lateral yeast emergence from hyphal filaments, as a key regulator of dispersal [8]. It was also demonstrated that dispersal from fungal biofilms depended on a balance between the yeast and hyphal population, wherein a larger population of filamentous forms resulted in corresponding lower frequencies of dispersal. This was evidenced by artificially manipulating master regulators of *C*. *albicans* filamentation such as *NRG1* and *UME6*, which showed that biofilm dispersal was inversely proportional to hyphal induction (and vice versa) [8,14]. Recent reports indicate that dispersal in *C*. *albicans* biofilms is also regulated by core members of a conserved histone deacetylase complex in *C*. *albicans* (Set3, Hos2, Snt1, and Sif2) that are additionally needed for proper biofilm formation and multifactorial drug resistance [15].

## What Are the Characteristics of Biofilm Dispersed Cells?

Whether dispersed cells display phenotypic and molecular characteristics similar to their source (the biofilm) or the population of cells they most appear like (planktonic cells) is a topic of active investigation. In order to colonize distal sites, cells released from biofilms must be able to disperse into the host environment and adhere to and damage the endothelial cells lining blood vessels before entering the tissues. In *C*. *albicans*, compared to free living cells, yeast cells dispersed from the biofilms are reported to be infectious particles displaying ~40% increase in both adherence (to plastic) and biofilm-forming ability [8]. Reinforced with properties of enhanced adhesion and filamentation, it was not surprising then to also find biofilm dispersed cells displaying enhanced adhesion and damage to endothelial cells, which signify major hallmarks of the infectious process [8]. Indeed, the cells dispersed from biofilms were significantly more lethal compared to their planktonic counterparts in a hematogenously disseminated murine model of candidiasis. This later finding indicates that dispersed cells may be able to retain their virulence properties over several generations, begging the question of if heritable epigenetic modifications are responsible for enhanced adhesion, filamentation, and virulence of dispersed cells.

For many years, the contribution to pathogenesis of dispersed cells from bacterial biofilms has mostly been discussed from a philosophical point of view. Few studies have actually examined the role of biofilm dispersal in virulence and manifestation of disease. Almost two decades ago, a study by Bieber et al., 1998, demonstrated that biofilm dispersal is required for full virulence of enteropathogenic *E*. *coli* in humans [16]. Using a model measuring diarrhea following oral inoculation in human volunteers, it was found that a mutant strain deficient in the production of type IV bundle-forming pili, which are required for biofilm dispersal, was 200-fold less virulent than a wild-type strain. A recent elegant report by Chua et al., 2014, presented the use of single-nucleotide resolution transcriptomic analysis to show that the physiology of dispersed cells from *P*. *aeruginosa* biofilms is very distinct from both planktonic and biofilm cells [17]. They found that expression of the small regulatory RNAs *RsmY* and *RsmZ* is down-regulated exclusively in the dispersed cell population, whereas secretion genes are induced. In the first series of experiments looking directly at virulence, they unveiled that dispersed cells are highly virulent against macrophages and *Caenorhabditis elegans* compared to conventionally grown planktonic cells. Finally, they described that the dispersed cell population is hypersensitive to iron stress, and thereby, combining an iron chelator with an antibacterial tobramycin is an effective therapeutic option against this cohort of cells.

## How is Biofilm Dispersal Modulated in Mixed-Species Biofilms?

The phenomenon of dispersion has mostly been studied in monospecies cultures, and extremely limited data is available on dispersal as a means of competing with other biofilm-forming bacteria in a mixed biofilm context. Some molecules involved in dispersion have a wide spectrum of activity against biofilms formed by other bacteria (discussed in the next section), and interestingly, these may also be used to outcompete other bacteria within a biofilm. For instance, a short-chain fatty acid signaling molecule, cis-2-decenoic acid, produced by *P*. *aeruginosa*, was found to disperse *Klebsiella pneumoniae*, *E*. *coli*, *Bacillus subtilis*, *S*. *aureus*, and even *Candida* biofilms in competition experiments [18].

Biofilms formed by organisms belonging to two different kingdoms (bacteria and fungi) are even more complex interactions, indulging a number of symbiotic and competing factors for sustenance together in a sessile condition. The best-studied examples of cross-kingdom biofilms include the presence of the fungal spp. *C*. *albicans* with *P*. *aeruginosa* or the growth of this fungus with other bacteria, such as *S*. *aureus* or *Acenitobacter baumanii*. Based on physical interaction data, it has been documented that bacteria have a dominating presence in the biofilm, where fungal filaments (but not yeast forms) serve in fact as a nutrient source for the bacteria [19]. Apart from cell–cell contact, cross-talk between the two sets of organisms via signaling molecules creates a tug-of-war between the species for growth and survival within the biofilm milieu. For example, while the *P*. *aeruginosa* quorum-sensing molecule 3-oxo-C12-homoserine lactone blocks germination and promotes filaments to yeast induction in *C*. *albicans* (via Ras-cAMP pathway) [20,21], the quorum-sensing counterpart in *C*. *albicans*, farnesol, can inhibit growth and promote cell death in bacteria such as *A*. *baumanii* and *S*. *aureus* [22,23]. Dispersal of cells from these mixed-kingdom consortia is thus contingent upon the complex nature of the interaction of the organisms within the biofilm and is a topic currently open for investigation.

## Can the Process of Dispersal Be Harnessed to Combat Biofilm-Mediated Diseases?

The most anticipated outcome of research on biofilm dispersal is the development of novel therapeutic and prophylactic approaches for the prevention and treatment of biofilm infections. Consortia of bacterial cells within the biofilms are protected from environmental insult, and the best possible approach to combat them is to break, dissolve, or disperse them into a population of planktonic cells that would immediately lose properties of antibiotic resistance. By and large, it is accepted that dispersed cells are physiologically more vulnerable than their presence in a sessile community [24]. In fact, increased antibiotic susceptibility has been observed with most dispersal agents, including those produced industrially, such as Dispersin B and DNase I (discussed below).

Several agents, such as biofilm matrix-degrading enzymes, quorum-sensing molecules, and inhibitors of diguanylyl cyclases (regulators of the important c-di-GMP), have been proposed for combating biofilms. Among the matrix-degrading enzymes, Dispersin B of Actinomyces *actinomycetemcomitans* has been shown to inhibit biofilm formation, induce biofilm detachment, and sensitize biofilms to killing by antibiotics and host defenses [25]. This molecule is a classic “dispersin” in that it solely induces biofilm disintegration without killing or inhibiting bacteria, and in doing so, it makes the bacterial cells more available for antimicrobials and simultaneously reduces the potential for emergence of drug resistance. Dispersin B treatment has also been shown to be effective against *Staphylococcus* biofilms [26]. Most recently, another enzyme, DNase I, has gained attention as a potential antibiofilm and pro-dispersal agent, particularly against gram-positive pathogens like *Staphylococcus* and *Enterococcus*, making these organisms vulnerable to biocides [27]. The effects of DNase I lie in its ability to digest the extracellular DNA (eDNA) found within the biofilm structure (while the exact function of eDNA in biofilms is not clear yet, it is predicted to have roles as a structural component, a nutrition source, or a gene pool for horizontal gene transfer) [4].

As discussed earlier, the most potent signal for bacterial biofilm dispersal is c-di-GMP. In a recent study, Ma et al. used a knockout library of previously uncharacterized genes known to be influenced by impaired autoinducer-2 secretion to identify BdcA as a protein that enhances biofilm dispersal by sequestering c-di-GMP and reducing its local concentration [28]. Indeed, the complex regulatory pathway of c-di-GMP, along with the diguanylyl cyclases that activate it, currently represents the most sought-after targets for development of novel antibacterial drugs [29]. Another powerful trigger for dispersal, NO, is being exploited as a pro-dispersal substance: using low concentrations of NO donor sodium nitroprusside (SNP), researchers were able to induce massive dispersal from *P*. *aeruginosa* biofilms, leading to 80% reduction in the amount of biomass on a glass surface [30]. Natural dispersion factors have also been exploited to dissociate gram-positive biofilms, in which exogenous addition of d-amino acids triggers biofilm dispersal in *B*. *subtilis* via detachment of amyloid fibers from the membrane [31]. In addition, d-amino acids have been shown to inhibit biofilm formation by *S*. *aureus* by inhibition of protein localization mechanisms [32].

While dissolution of biofilms represents an attractive approach to curtail biofilm-associated infections, a consensus has not been reached so far on how dispersal could be targeted in fungal biofilms. This is basically due to the lack of systematic studies on the phenomenon to lead to a rational agreement. Fungal biofilms are almost totally made up of a complex mesh of hyphae and glued together with abundant EPS. This gives the biofilm its robust form that is extremely tough to dissociate, even by external treatments. While a classical “dispersin” has not been identified for fungal biofilms, DNase treatment of *C*. *albicans* biofilms has been found to cause significant biofilm disintegration (in mature biofilms only) by perhaps acting on the biofilm matrix rather than cell viability [33]. Nevertheless, how breaking up chunks of biofilm hyphae or detachment of thick fungal biofilms will translate into better therapeutic outcomes remains to be investigated.

An alternative school of thought is that, since dispersal in fungi such as *C*. *albicans* is initiated from the hyphal layers of the biofilm, one way to contain biofilm-mediated infections is perhaps to inhibit dispersal from biofilms. Unfortunately, caspofungin (from the drug class echinocandins) was found to be the only drug that could deactivate biofilm dispersal in *C*. *albicans* within a clinically acceptable concentration range [34]. The other two drugs tested, fluconazole (an azole) and amphotericin B (a polyene) had minimal effects on prevention of release of cells from the biofilm. Further, only caspofungin had a potent killing activity on these dispersed cells when compared to age-matched planktonic cells using CLSI (Clinical and Laboratory Standards Institute) guidelines. In fact, dispersed cells were found to be four times more resistant to fluconazole [34].

Since none of the currently available classes of drugs (perhaps with the exception of echinocandins) inhibit biofilms or biofilm dispersal, there is an urgent need to discover newer antifungal molecules that can perhaps do both. An example of one such class of molecules is fungal Hsp90 inhibitors, which were found, along with therapeutic concentrations of fluconazole, to not only completely kill fungal biofilms (including *C*. *albicans* and *A*. *fumigatus*) but also on their own render >90% of the biofilm dispersed cells inviable [35].

## Future Directions

A major driving force behind studying the biofilm life cycle is to identify and develop novel targets that have the potential to be interjected by inhibitory molecules. The mechanisms underlying dispersal from biofilms are excellent targets because, in bacteria, induction of the process triggers an intriguing reversal of the gene expression program, causing dissolution of biofilms followed by hypersusceptibility of the dispersed population to antimicrobial drugs. In fungi, inhibition of that very program, which is key for dispersal, is important to manage biofilm-mediated adverse effects. The future of biofilm management also lies in the discovery of novel molecules that can interfere with the broad intracellular signals and regulatory systems controlling dispersal in order to ameliorate biofilm-associated problems.

Finally, as a result of innumerable factors, there is a high degree of complexity that distinguishes biofilms in vivo from those formed in vitro, including environmental parameters, interaction with host cells, and presence of other species of commensal microbes. What impact these factors have on dispersal in vivo is still an enigma. An integration of knowledge gained from in vitro studies on biofilms, together with a better understanding of the biofilm dispersal signatures in vivo, will likely represent a key requirement for the control of biofilm-mediated diseases in humans.
